# Das Höhenklima in meteorologischer, physiologischer und therapeutischer Beziehung

**Published:** 1889-06

**Authors:** 


*Das Höhenklima in meteorologischer, physiologischer und therapeutischer Beziehung.* Von Dr. AUGUST LADENDORF. Berlin: EUGEN GROSSER. 1889.

The first part of this little book has reached us, and in it the author deals with the meteorological conditions of elevated regions in different parts of the world. He treats most elaborately of the power of the sun, the amount of sunshine per year, and the amount of mist brought up by it; also, the direction of prevailing winds, their force and their effect on temperature and rainfall, are set forth in tabular form.

